# Contrasting the Expectations and Experiences Related to Mobile Health Use for Chronic Pain: Questionnaire Study

**DOI:** 10.2196/38265

**Published:** 2022-09-06

**Authors:** Saba Kheirinejad, Andy Alorwu, Aku Visuri, Simo Hosio

**Affiliations:** 1 Center for Ubiquitous Computing University of Oulu Oulu Finland

**Keywords:** mobile health, mHealth, m-Health, mhealth, m-health, wearable devices, mobile apps, self-management, digital health, chronic pain, pain, wearable, questionnaire, crowdsourcing, crowdsourced, user feedback, usage pattern, patient feedback, perception, attitude, user experience

## Abstract

**Background:**

Chronic pain is a prolonged condition that deteriorates one's quality of life. Treating chronic pain requires a multicomponent approach, and in many cases, there are no “silver bullet” solutions. Mobile health (mHealth) is a rapidly expanding category of solutions in digital health with proven potential in chronic pain management.

**Objective:**

This study aims to contrast the viewpoints of 2 groups of people with chronic pain concerning mHealth: people who have adopted the use of mHealth and those who have not. We highlight the benefits of mHealth solutions for people with chronic pain and the perceived obstacles to their increased adoption. We also provide recommendations to encourage people to try mHealth solutions as part of their self-care.

**Methods:**

The Prolific crowdsourcing platform was used to collect crowdsourced data. A prescreening questionnaire was released to determine what type of pain potential participants have and whether they are currently using mHealth solutions for chronic pain. The participants were invited based on their experience using mHealth to manage their pain. Similar questions were presented to mHealth users and nonusers. Qualitative and quantitative analyses were performed to determine the outcomes of this study.

**Results:**

In total, 31 responses were collected from people (aged 19-63 years, mean 31.4, SD 12.1) with chronic pain who use mHealth solutions. Two-thirds (n=20, 65%) of the users identified as female and 11 (35%) as male. We matched these mHealth users with an equal number of nonusers: 31 responses from the pool of 361 participants in the prescreening questionnaire. The nonusers’ ages ranged from 18 to 58 years (mean 30.8, SD 11.09), with 15 (50%) identifying as female and 15 (50%) as male. Likert-scale questions were analyzed using the Mann-Whitney-Wilcoxon (MWW) test. Results showed that the 2 groups differed significantly on 10 (43%) of 23 questions and shared similar views in the remaining 13 (57%). The most significant differences were related to privacy and interactions with health professionals. Of the 31 mHealth users, 12 (39%) declared that using mHealth solutions has made interacting with health or social care professionals easier (vs n=22, 71%, of nonusers). The majority of the nonusers (n=26, 84%) compared with about half of the users (n=15, 48%) expressed concern about sharing their data with, for example, third parties.

**Conclusions:**

This study investigated how mHealth is currently used in the context of chronic pain and what expectations mHealth nonusers have for mHealth as a future chronic pain management tool. Analysis revealed contrasts between mHealth use expectations and actual usage experiences, highlighting privacy concerns toward mHealth solutions. Generally, the results showed that nonusers are more concerned about data privacy and expect mHealth to facilitate interacting with health professionals. The users, in contrast, feel that such connections do not exist.

## Introduction

### Background

Pain is chronic when it lasts more than 12 weeks despite therapy and medication after any initial injuries and when underlying causes have been treated [1]. Various chronic pains are extremely burdensome worldwide and severely detrimental to the quality of life. For instance, roughly 20%-25% of the adult population (between 20 and 59 years old) develops chronic low back pain (CLBP) symptoms at some point in their lives [2]. Treatment of chronic pain is complex and requires a multicomponent approach, which may not always be available [3]. No “silver bullet” solutions to chronic pain exist. Thus, there is always a need to explore potential new ways to alleviate pain symptoms or to improve the quality of life in other ways for people with chronic pain. To this end, and specifically relevant to the human-computer interaction (HCI) community, mobile health (abbreviated as mHealth, mhealth, m-health, or m-Health) is a rapid concept in the field of digital health.

In general, mHealth is defined as medical or public health practice supported by mobile devices [4] and contains a variety of contexts, such as the use of mobile phones to the point of service data collection, care delivery, patient communication, use of alternative wireless devices for real-time medication monitoring, and adherence support [5]. mHealth is not only a solution and tool for personal usage but in many countries has also been adopted in different health care places, such as hospitals and clinics. It is a good solution and tool to collect and provide various types of information about patient health and vital status to medical providers [6]. Most mHealth solutions gather data about a person’s physiology, physical activity, or social behavior and are designed to keep the data for later analysis by providers and caretakers [7]. In other words, mHealth solutions can potentially serve as a complementary tool for collecting, analyzing, and presenting data to users or health professionals to aid their understanding of users’ health and well-being. Smartphones are currently the most popular platform for mHealth delivery [8]. Recent explorations have also started to investigate mHealth for pain management [9]. A preliminary study proposed that mHealth self-management methods, such as mobile apps, could manage chronic pains, such as CLBP, better than only physiotherapy [10], especially since the beginning of 2020 when COVID-19 has globally become prevalent and its impact might last until 2025 [11]. COVID-19 has led to the rapid development of mHealth solutions [12]. Currently, many back pain apps are available in different stores focused on pain management education [13]. App-based solutions are almost available 24/7 and do not have geographical limitations for people from rural or remote areas [14]. Approaches including education, advice, and a major focus on self-management, such as lifestyle change, physical activity, and medications, as required, could be adopted to lift the burden of treatment off the clinicians and help the patients self-manage their pain [15].

In this paper, we set out to understand what people with chronic pain think about mHealth from 2 complementary perspectives. First, we explored how people with chronic pain experience mHealth solutions. Second, we explored the prevailing expectations toward mHealth by people with chronic pain who do not use mHealth solutions. To this end, we deployed a series of online questionnaires to the crowdsourcing and crowdworking platform *Prolific*. We analyzed responses from 62 participants with chronic pain: 31 (50%) mHealth users and 31 (50%) nonusers. The key contributions in the context of mHealth and chronic pain in our work are as follows:

- We present an overview of user experiences with mHealth, including perceived benefits, obstacles, and practical usability matters.
- We match the overview of user experiences with a corresponding account of expectations toward mHealth from people who have not adopted mHealth devices.
- Finally, based on our results, we highlight implications for mHealth solutions to manage chronic pain.

As a result of the presented data analysis, this study helps understand the future role of mHealth in chronic pain management. This includes an account of the benefits such technology should offer to become more prevalent and an exploration of why people seem to opt out of this potentially beneficial class of advanced health technologies. Put together, our insights have implications for mHealth designers and researchers in the form of topical issues to address and research avenues to explore.

### mHealth

For more than a decade, mHealth has been suggested to improve health care systems and delivery services, although it should be noted that there is no standard and universally accepted definition for mHealth in the research literature. The term “mHealth” was first coined in 2003 and is defined as “mobile computing, medical sensor and communications technologies for health care” [16]. Free et al [17] defined mHealth as “the use of mobile computing and communication technologies in health care and public health.” In another study, mHealth was defined as “a subset of e-health using mobile devices to deliver health services to patients” [18]. The World Health Organization (WHO) Global Observatory for eHealth (GOe) [4] defines mHealth as “medical and public health practice supported by mobile devices.”

Over time, the definition of mHealth has been changing as new technologies have emerged, and recent studies have presented a clearly broader concept for mHealth. More recent studies consider wearable sensors as mHealth solutions [19-21]. At the same time, based on the definition of WHO, other wireless devices could also be interpreted as wearable sensors. These include, for example, smartwatches that connect to wireless networks. It should be noted that the notion of wearable sensors has a tight association with mobile apps. In other words, mobile apps practically always accompany wearable devices, and it is difficult to separate them from smartphones, because of the ambiguous nature of mobile technology. In the same vein, Istepanian et al [16] presented the architecture of mHealth with 3 building blocks: computing and the internet (eg, artificial intelligence [AI], cloud, and big data), communication systems (eg, 5G and internet of things [IoT]), and sensors (eg, body area network [BAN], personal area network [PAN], and tactile). Later, Istepanian [22] considered medical apps, wearable sensors, and mobile devices as integral parts of mHealth service architecture.

The global adoption of mHealth is growing due to decreasing hardware costs and the increasing amount of, for example, smartphones, tablets, and wearable devices in circulation [23]. The services provided by mHealth solutions also help global adoption of mHealth, such as numerous health applications that encourage healthy lifestyles by assisting users in exercising regularly or monitoring their heart rate, measuring step numbers, etc [24,25]. The factors toward the adoption of mHealth apps among adults are relative advantage, ease of use, and compatibility [26]. Moreover, mHealth apps in developing countries are considered one of the best platforms for ensuring the citizens' safety and health care security [27]. In general, the factors behind adopting mHealth solutions could include performance, effort expectancy, social influence, hedonic motivation, price value, habit, facilitating conditions, privacy, lifestyle, self-efficacy, and trust [28]. The number of connected wearable devices worldwide more than doubled in the space of 3 years, increasing from 325 million in 2016 to 722 million in 2019. The number of such devices was anticipated to grow to more than 1 billion by 2022 [29]. On the software side of things, an estimation 100,000 apps on Google Play Store and Apple Store combined belong to the medical, health, fitness, and wellness categories [30].

mHealth solutions are commonly used in public health care and health services, where they are appreciated for their ease of use, broad reach, and wide acceptance [31]. mHealth has also been shown as beneficial, for example, in rural areas, for the overall development of health care systems [23,32]. As mHealth solutions have become more accessible, their use has been steadily increasing among laypeople as well [33]. Here, purposes include helping people succeed in weight management, stress management, smoking cessation [34]; encouraging and monitoring behavior change, self-diagnosis, and rehabilitation schedule management [35]; and self-monitoring chronic health conditions, medicine adherence reminders, and direct interactions with the health care system [36].

mHealth may also enable meaningful information exchange between consumers and health care professionals. mHealth services can collect and distribute electronic records, patient data, remote monitoring, and electronic prescriptions, or fitness and wellness apps can provide supplementary data to caretakers, for example [37]. Yet, medical experts are still somewhat reluctant to use mHealth solutions as part of their treatment, due to insufficient evidence of their benefits [5]. The key to developing mHealth in a beneficial direction for all stakeholders is cooperation. Indeed, the development of mHealth solutions requires a diverse set of expertise, including software programmers, behavioral scientists, graphical designers, and medical experts, such as doctors and physiotherapists. It also requires end-user feedback about the solutions so that the vendors can match actual users’ real-life needs [38,39].

### Chronic Pain Management Using mHealth

mHealth solutions are rated valuable and easy to use by patients living with chronic pain [3,40]. Adherence to medication and treatment is essential in pain management. mHealth has been shown as applicable to encourage people to continue treatment after being discharged from clinical care [40]. Cheong et al [41] showed an improvement in physical performance, cancer alleviation, and symptoms related to cancer treatment for patients with colorectal cancer undergoing chemotherapy using mHealth and the IoT. Buneviciene et al [42] evaluated mHealth solutions, such as activity/fitness, cognitive behavioral therapy, and mindfulness/stress management interventions, for cancer patients. They showed that mHealth solutions could improve the health-related quality of life of patients with cancer [42]. Hosio et al [43] developed a crowdsourced online system named *Back Pain Workshop*. They collected 2 knowledge bases, 1 from clinical professionals and 1 from nonprofessionals. Professionals found the system beneficial for self-reflection and educating new patients, while nonprofessionals acknowledged the reliable decision support that also respected the nonprofessional opinion [43]. Monitoring hospitalized patients’ pain is a crucial problem for clinical caregivers, although collecting pain reports from patients can be challenging and time-consuming for clinicians. Price et al [44] provided a tangible device named *Painpad*, which allows patients to self-log their pain. They found that the self-logged scores might be more faithful than those reported to nurses. They also showed that older adults might prefer tangible interfaces over tablet-based alternatives for reporting their pain [44].

Given how smartphones have grown in recent years, mobile app–based self-management has become prevalent. Studies indicate that app-based therapy can benefit pain reduction, especially when practiced long term [14]. Smartphone app–based self-management programs have been developed to improve the physiotherapy status of patients with CLBP [10]. mHealth-based exercises are a valuable and efficient method to improve back pain [45]. Bailey et al [46] conducted a longitudinal observational study on a large population of patients with CLBP using a mobile app. Participants illustrated a high engagement rate in the study, and the results showed a significant positive relationship between engagement and pain decrease [46]. Hourcade et al [47] presented a zoomable multitouch app to enable children with a chronic headache to draw their symptoms on it during medical appointments. The app gives them the ability to provide more details and context than on paper. They showed that the app helps children better communicate their symptoms, and health care professionals can also better treat them [47]. Adams et al [48] investigated how those living with chronic pain prefer to self-assess their pain levels using smartphones. They developed a novel smartphone-based assessment tool and focused on designing visual interfaces for self-reporting pain intensity on smartphone screens [48]. Nevertheless, despite a perceived high demand from physicians, there are not enough suitable pain apps for clinical usage [49].

Rodríguez et al [50] showed that over two-thirds of people prefer the wearable option when they are given the choice between a wearable device and a mobile app for self-reporting pain. Adams et al [51], motivated by the need to manage chronic pain, reported a new pressure-based tangible user interface (UI) for the self-reporting of pain intensity, named *Keppi*. They also created wearable versions of Keppi, such as necklaces, bracelets, and keychains [51]. Cuia et al [52] presented a smart baby carrier connected to a digital frame named *CarryLine* to manage postnatal chronic back pain and rehabilitate patients. CarryLine encourages physiotherapist-recommended activity in an innovative and engaging way [52].

Chronic pain reveals many different forms and imposes extensive physical restrictions on the patient’s body. However, this pain is invisible and incommunicable, and it becomes complicated for the public to understand or even believe the patient, especially the persistent kind of pain. Therefore, the mental and social problems related to chronic pain are often neglected. Jin et al [53] developed a game called *AS IF* to increase nonpatients’ empathy for those with chronic pain. In this game, after players connect to their virtual body, they experience a specific level of activity limitation that imitates one of the difficulties due to chronic pain [53]. Shah [54] provided a gameplay tool named *On the Other Side* and made the players aware of the troubled life of a patient with chronic pain.

## Methods

### Study Design

We set out to investigate how mHealth solutions are used and experienced. To this end, we wanted to explore what expectations and assumptions mHealth nonusers have about the technology and how far those assumptions are from the actual experiences of people who have adopted mHealth already. We used the Prolific human subjects pool to collect crowdsourced information, as it combines good recruitment standards with reasonable cost [55]. Prolific is widely used in behavioral research and questionnaire studies and provides data of high validity [56,57]. Prolific manages the privacy and anonymity of its participants through various policies (eg, a privacy policy and legal terms) that both the researchers using the platform and the participants must agree to prior to using the platform. It also meets the high standards of the European data protection law (General Data Protection Regulation [GDPR]) and is commonly used to recruit anonymous participants online. We focused our investigation specifically on a population that experiences chronic pain. Using the Prolific platform allowed us to prescreen the potential participants for those who have reported suffering from chronic pain.

### Ethical Considerations

Our study design followed the ethical procedures required by the host university ethics board (ie, an individual study does not require ethics board reviews as long as the study does not pose a significant risk of harm to the participants). Informed consent from the participants is sufficient for the type of study presented in our work [58].

### Prescreening Questionnaire

Our first step was to design a prescreening questionnaire (Multimedia Appendix 1) to determine what type of pain our potential participants have and whether they are currently using any mHealth solutions for their chronic pain. The prescreening questionnaire contained the following 4 questions:

- What type of chronic pain do you have? (Categorical listing of various types of chronic pain)
- How long have you suffered from it (in years)?
- How do you manage your pain in general? (Text field)
- Do you currently use any mHealth solutions related to your chronic pain (yes/no)?

Using this information, we then later invited participants, especially based on their answer to question 4 (whether they have experience with using mHealth to manage their chronic pain). Participants in this stage were compensated EUR 0.50 (US $0.58) for their responses, which typically only took a few minutes to complete. The prescreening questionnaire was not piloted before deployment.

We invited 400 participants with chronic pain by using the prescreening options of Prolific. Participants’ ages ranged from 18 to 74 years (mean 28.7, SD 10.3), with 234 (58.6%) identifying as female and 164 (41.1%) as male (demographic information of participants was missing). Of the 400 participants, 39 (9.7%) indicated currently using mHealth solutions and 361 (90.3%) revealed that they do not use mHealth. We selected all the 361 mHealth nonuser participants.

### Questionnaire Design

The use of technology is driven by trust and how effectively the technology meets the expectations that its user associates with it. Continued use of potentially useful applications and systems is not always a given, and abandonment of, for example, wearable technologies is a common problem [59]. We designed our investigation based on the expectation disinformation theory (EDT) model [60], with the trust-in-technology concept brought into the model. The EDT model is based on expectations (pre-exposure) and perceived performance (postexposure). Postexposure can *disconfirm* technology expectations, which leads to usage satisfaction and continued use. The theory has been widely used in different contexts, ranging from trust toward digital assistants [61] and unfamiliar online information sources [62], which is something that can often be part of mHealth solutions you are unfamiliar with, and trust toward treatment methods for back pain, for example [63]. Because our questionnaire is not a longitudinal process that incorporates information from a set of users over a period, we relied on generalizing results from 2 subgroups of participants based on our prescreening questionnaire. The mHealth *users* represented the *postexposure* participant pool according to the EDT model, while the mHealth *nonusers* represented the *pre-exposure* participants. However, the same participants for both groups were not used, as it was not optimal in terms of time and facilities to use the same participants for pre-exposure and postexposure groups in this study, although in a nonlongitudinal survey study, the methodology should be valid (comparing 2 groups should be fine).

We investigated related standardized questionnaires to complement the EDT and to ensure appropriate language and framing of each question. Fred [64] designed questions for perceived usefulness, ease of use, and user acceptance of information technology. Lund [65] developed the Usefulness, Satisfaction, and Ease of use (USE) questionnaire to measure the usability of UIs. Kortum and Sorber [66] measured the usability of mobile apps for phones and tablets using the System Usability Scale (SUS) questionnaire, which has 10 questions that investigate the ease of use and learning and the functionality of the apps. Parmanto et al [67] developed the Telehealth Usability Questionnaire (TUQ) to measure the quality of telehealth interaction and services and the computer-based UI. The TUQ includes 6 categories: usefulness, ease of use, and learnability; interface quality; interaction quality; reliability; satisfaction; and future use [67]. Measuring the acceptability of telehealth users can afford valuable information to services to increase telehealth use. Hirani et al [68] reported developing and validating the Service User Technology Acceptability Questionnaire (SUTAQ). SUTAQ is a tool to measure the acceptability of telehealth, quality of life, well-being, and psychological conditions of the users. SUTAQ scales include increased accessibility, privacy and discomfort, care for personal concerns, telehealth as a substitution, and satisfaction [68]. Reicher et al [69] investigated the adults’ attitude toward telemedicine during COVID-19 lockdown using an online questionnaire. They investigated 5 items: the necessity of using telemedicine, satisfaction with it, willingness to use it, change of mind regarding it, and preference to use it rather than going to a clinic [69]. Yen et al [70] developed the Health Information Technology Usability Evaluation Scale (Health-ITUES) questionnaire by asking nurses to rate the usability of a web-based communication system for scheduling nursing staff. The Health-ITUES has 4 categories: quality of work-life, perceived usefulness, perceived ease of use, and user control [70].

Regarding the questionnaire design, we used standardized questionnaires as a basis to seek questionnaire items that are both understandable and domain relevant. We also wanted to explore the topic from multiple viewpoints without overburdening the respondents with too many questionnaires. However, our questionnaires are not intended to be a standardized questionnaire to be used across all of mHealth; they were constructed for the purposes of this study only.

### Finalized Questionnaire Based on Related Research

Incorporating the aforementioned literature, as well as privacy elements, our final adapted and extended EDT-based questionnaire themes were (1) ease of use, (2) functionality, (3) reliability, (4) usefulness, (5) other expectations and impressions, and (6) privacy. The 2 questionnaires used the same themes to contrast expectations (nonusers) and experiences (users). Still, the questions are framed to be either about the participants’ expectations *toward* mHealth or the participants’ experiences *with* mHealth. The full questionnaires include 22 Likert-type items, 13 open-ended questions, and a single multiple-choice question. The Likert items were articulated using a consistent 1-7-point “not at all” to “extremely” wording scheme, depending on the specific item. The complete questionnaire items can be found in Multimedia Appendices 2 and 3. Additionally, the complete list of topics covered in the questionnaire, along with references from which they were derived, can be seen in Table 1.

**Table 1:** Subthemes and Likert item topics in the 2 questionnaires.

Subtheme	Likert item topics
Ease of use	E1: easy to use [64-67,70-73] E2: easy to learn [64-67,70-73] E3: easy to become skillful with it [64,65,67,70]
Functionality	F1: features and functions fulfil expectations [71]
Reliability	R1: reliable [71,72] R2: source credibility concern [71]
Usefulness	U1: useful [64,65,70] U2: data used by doctors in office visit [72] U3: supports routine adherence [72] U4: reduces concern about chronic pain [68] U5: aids chronic pain management [68,72,73] U6: saves time [65,67,68,72,74] U7: control over one’s life [64,65,73] U8: supports interaction with medical staff [67,68,72,74]
Other expectations and impressions (satisfaction)	O1: future use [67,74] O2: fun to use [65,75] O3: recommend to others [65,68]
Privacy	P1: invades privacy [73,76] P2: donate data for additional features [77] P3: data access [77] P4: data sharing [30,35,77]

### Questionnaire Deployment and Participant Overview

We invited the 39 participants who indicated that they used mHealth to complete the follow-up questionnaire and received 26 (67%) responses from the prescreening questionnaire. We conducted a small-scale supplementary data collection using our university mailing lists by adding demographic data questions, which were included by Prolific in the first batch of data. This led to 5 more submissions: in total, we collected 31 responses from people with chronic pain who use mHealth solutions. These participants’ ages ranged from 19 to 63 years (mean 31.4, SD 12.1), with 20 (65%) identifying as female and 11 (35%) as male. Of 31 mHealth users, 15 (48%) indicated using a smartwatch, 20 (65%) used mobile apps, 8 (26%) used activity trackers, 1 (3%) used a Holter heart monitor, and 1 (3%) used an oximeter.

Subsequently, to match these mHealth users with an equal number of nonusers, we released the second version of the questionnaire about expectations to obtain 31 (8.6%) responses from our pool of 361 participants who indicated not using mHealth solutions in the prescreening questionnaire. Reaching out to people with chronic pain who used mHealth from a pool of 39 persons was more challenging than reaching out to nonusers from a pool of 361 (of 400 people who had chronic pain, only 39, 9.7%, persons said that they use mHealth and 361, 90.3%, persons answered that they do not use mHealth). Hence, we first had to determine how many users out of 39 we could reach, and then we hired the same number of nonusers to have a fair comparison. Nonusers’ ages ranged from 18 to 58 years (mean 30.8, SD 11.9), with 15 (50%) identifying as female and 15 (50%) as male (demographic information of participant was missed in Prolific). In addition, 2 (6%) of the 31 participants had previously used mHealth solutions but did not use one currently.

We compensated the participants with EUR 5.00 (US $5.91) for their responses.

## Results

### Data Analysis

We presented similar questions to the 2 groups, *mHealth users* and *mHealth nonusers*, with slight modifications. mHealth users were asked to offer their opinions based on their experiences, while nonusers were asked their expectations. Examples of questions that were asked of both groups with slight modifications are as follows:

- “How easy did you find the use of the mHealth solution(s) that you use?” (for users) versus “How easy would you expect the use of the mHealth solution(s) to be?” (for nonusers)
- “How easy did you find learning to use the mHealth solution(s) that you use?” (for users) versus “How easy would you expect learning to use the mHealth solution(s) to be?” (for nonusers)

We analyzed the Likert-type questions using the Mann-Whitney-Wilcoxon (MWW) test, a nonparametric test that checks whether 2 samples are derived from a similar population.

**Figure 1:**
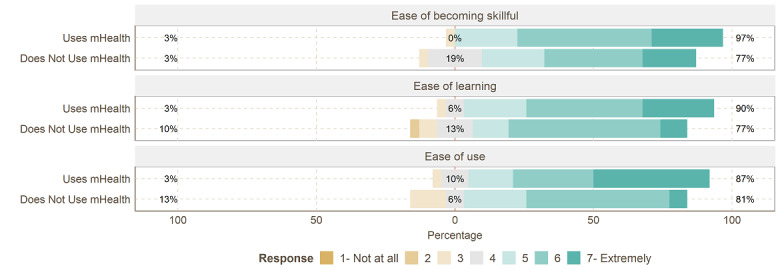
Likert item answers to ease of use. mHealth: mobile health.

**Figure 2:**
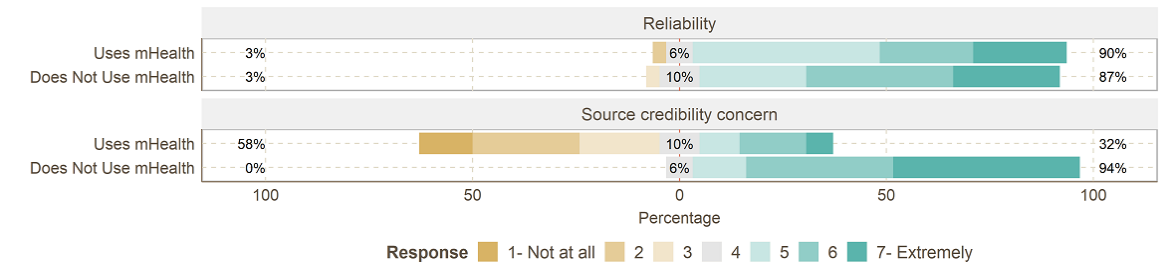
Likert item answers to reliability. mHealth: mobile health.

**Figure 3:**
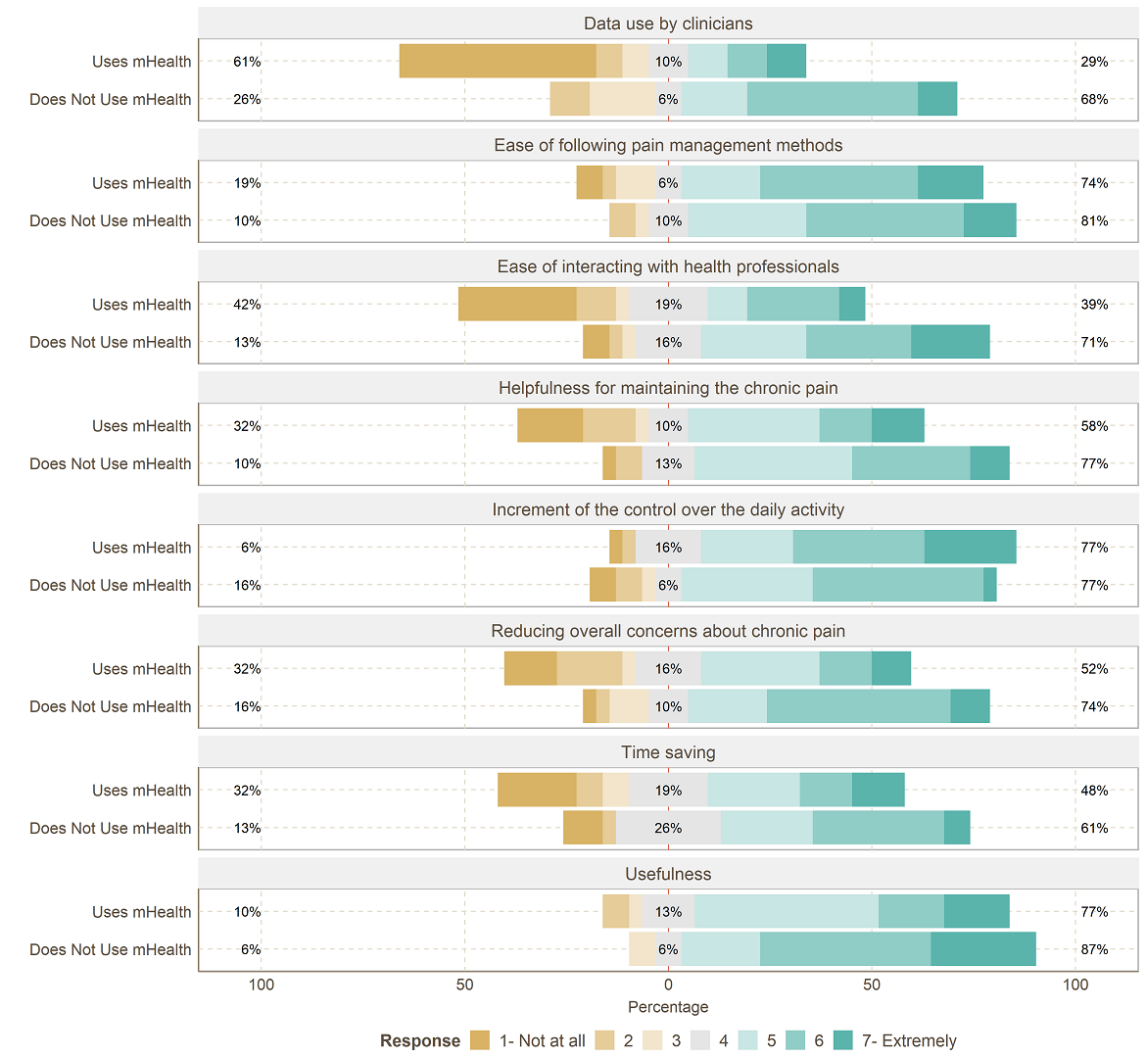
Likert item answers to usefulness. mHealth: mobile health.

**Figure 4:**
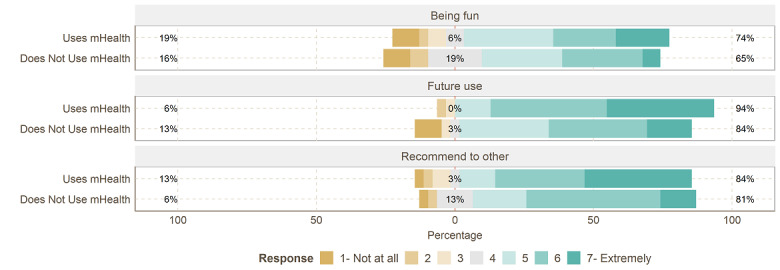
Likert item answers to other expectations and impressions. mHealth: mobile health.

**Figure 5:**
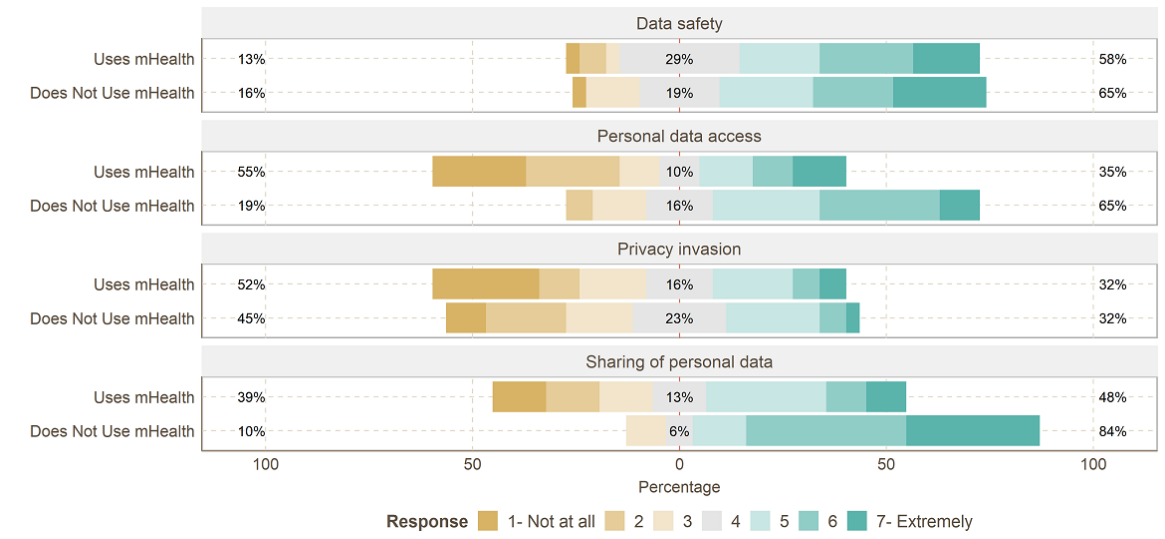
Likert item answers to privacy. mHealth: mobile health.

The qualitative data were analyzed following the directed content analysis method [78] based on individual questionnaire items. Our analysis was conducted with specific questions in mind. Due to the COVID-19 pandemic, the authors held multiple online meetings to discuss and resolve any disagreements that emerged. Our results showed that the 2 groups had a significantly differing stance on 10 (43%) of the 23 Likert-type questions (described in detail in the following sections), as seen in Table 2 and Figures 1-5*.* However, they tended to share similar views in the remaining 13 (57%) questions.

**Table 2:** MWW^a^ statistical test results for Likert-type questions (neutral response=4 on a scale of 1-7).

Question			User mean (SD)	Nonuser mean (SD)		*P* value
**Ease of use**						
	E1: How easy (did you find/would you expect) the use of mHealth^b^ solution(s) (that you use/to be)?	5.96 (1.13)		5.32 (1.13)	*.01* ^c^	
	E2: How easy (did you find/would you expect) learning to use mHealth solution(s) (that you use/to be)?	5.80 (1.01)		5.38 (1.22)	.20	
	E3: How easy (was it for you/would you expect) (to become/becoming) skillful at using mHealth solution(s) (that you use/to be)?	5.93 (0.89)		5.48 (1.12)	.10	
**Reliability**						
	R1: How reliable (do you find/would you expect) mHealth solution(s) (that you use/to be) in general?	5.51 (1.12)		5.70 (1.07)	.44	
	R2: How concerned (are you/would you expect to be) about the source credibility of mHealth solutions?	3.51 (1.89)		6.19 (0.90)	*<.01* ^c^	
**Usefulness**						
	U1: How useful (do you find/would you expect) mHealth solutions (/to be) for tracking or managing your chronic pain in general?	5.09 (1.30)		5.74 (1.12)	*.02* ^c^	
	U2: How much (does/would you expect) your doctor (/to) use information from your mHealth solution(s) during office visits?	2.93 (2.24)		4.93 (1.56)	*<.01* ^c^	
	U3: How much easier (is it/would you expect it to be) to follow medical advice, treatment guidelines, or any potential exercise routine (you are following since starting to use/if you used) mHealth solutions?	5.09 (1.70)		5.29 (1.29)	.97	
	U4: How helpful (did you find/would you expect) mHealth solutions (/to be) in reducing your overall concern about your chronic pain?	4.09 (1.90)		5.12 (1.47)	*.01* ^c^	
	U5: How much (do/would you expect) mHealth solutions (/to) help you in maintaining your chronic pain?	4.19 (2.02)		5.03 (1.40)	.11	
	U6: How much time, if any, (do you/would you expect to) save because of using mHealth solutions?	4.09 (2.02)		4.70 (1.63)	.23	
	U7: How much easier (have/would you expect) mHealth solutions (made/to make) it to interact with health or social care professionals?	3.64 (2.15)		5.06 (1.65)	*.01* ^c^	
	U8: How much more control (do/would you expect) mHealth solutions (/to) give you over the activities in your life?	5.38 (1.45)		4.90 (1.55)	.21	
**Other expectations and impressions**						
	O1: How much (are you planning/would you expect) to use mHealth solutions in the future?	6.03 (1.16)		5.19 (1.66)	*.01* ^c^	
	O2: How fun (did you find/would you expect) mHealth solutions (/to be)?	4.93 (1.80)		4.64 (1.68)	.38	
	O3: How much would you (/expect to) recommend mHealth solutions to other people who are in a similar situation to you (they also have chronic pain)?	5.70 (1.59)		5.38 (1.35)	.11	
**Privacy**						
	P1: How much (do you think/would you expect (anticipate)) mHealth solutions (/to) invade your privacy?	3.38 (1.90)		3.61 (1.58)	.57	
	P2: In your estimate, how safe (are/would you expect) your data collected through your mHealth solutions (treated/to be treated)?	4.87 (1.56)		5.06 (1.54)	.65	
	P3: How concerned (are you/would you expect to be) about your mHealth solution manufacturer having access to your personal data collected via the mHealth solution?	3.48 (2.14)		4.87 (1.40)	*<.01* ^c^	
	P4: How concerned (are you/would you expect to be) about your personal data being shared with, for example, third parties without your permission?	4.00 (1.86)		5.77 (1.25)	*<.01* ^c^	
**I (would be/would expect to be) willing to share the following information.**						
	First name	4.80 (2.21)		4.96 (1.94)	.95	
	Last name	4.19 (2.38)		4.29 (2.19)	.95	
	Email address	4.58 (2.09)		4.41 (2.02)	.75	
	Phone number	2.61 (1.89)		2.74 (1.71)	.63	
	Residential address	1.41 (0.80)		2.09 (1.57)	.06	
	Iris pattern	1.61 (1.33)		1.90 (1.30)	.13	
	Fingerprint	1.22 (0.61)		1.38 (0.71)	.32	
	Birth date and national identification number	1.80 (1.51)		2.58 (1.64)	*.03* ^c^	
	Debit or credit card	1.45 (0.99)		1.32 (0.65)	.95	
	Location data	2.45 (1.82)		2.51 (1.76)	.87	

^a^MWW: Mann-Whitney-Wilcoxon.

^b^mHealth: mobile health.

^c^All italicized *P* values are significant.

### Ease of Use

We asked participants to share their experiences and expectations regarding the ease of use and learnability of mHealth solutions. mHealth nonusers expected mHealth apps to be easy to use, require less time, be fully automated, and not require looking up documentation. These expectations were matched by the experiences of mHealth users who expressed a positive attitude toward the ease of use of mHealth solutions in general, as summarized by 1 participant:

[mHealth solutions]...are nicely designed and completely intuitive.

User 18

As depicted in Figure 1, the nonusers’ expectations and users’ experiences did not differ much, as both groups considered *ease of use* an important factor for the adoption and use of mHealth solutions. We also observed a statistically significant difference between mHealth users’ experience of *ease of use* and nonusers’ expectations (mean 5.96, SD 1.13, vs mean 5.32, SD 1.13; *P*=.01). This is expected as nonusers cannot tell how easy something is to use when they have not used it.

Despite the reported ease of use of mHealth solutions among mHealth users, setting up of the mHealth solutions was not all smooth for some users, as they reported facing difficulties while getting started. These reported difficulties support a mixture of fear and skepticism expressed by some nonusers about the ease of use of mHealth solutions, with 1 nonuser stating:

...It won’t be a walk in the park.

Nonuser 31

However, we believe these fears are realistic, given that the learnability of any software or hardware tool sometimes takes time. To this end, some mHealth users shared some of the difficulties they faced in their early personal experiences with mHealth solutions:

My wearable tracker was difficult to set up initially, but once it was installed, it was very easy to use.

User 7

One participant was, however, quick to point out the availability of documentation to aid in the onboarding process:

...The instructions are always understandable (discussed step by step).

User 4

Concerning previous issues that might have affected the current nonusers’ decision to stop using mHealth solutions, the participants mentioned both hardware- and software-related issues, such as Bluetooth pairing between their hardware devices (eg, wearables) and their mobile phones, software bugs after updates, incorrect or poor translations, discomfort with wearing wearable devices, and software crashes. Similar sentiments were expressed by some current mHealth users as well, as 1 participant noted:

...Auto Bluetooth connectivity to the smartphone is not always great.

User 18

Only 1 mHealth user mentioned having had issues with the UI of an mHealth app:

...Mostly the issues I had were related to the UI not being the most easily understandable (ie, features were hidden in weird places or just hard to find).

User 31

This was interesting to find as most nonusers were particularly concerned about how mHealth solutions could be “intimidating or overwhelming for inexperienced tech users” (nonuser 18).

### Functionality

mHealth nonusers outlined various functions they would love mHealth solutions to perform for them. Most mHealth nonusers perceived mHealth solutions as telemonitoring apps that would enable medical staff to monitor them constantly and help patients access help from these health professionals when they feel the need to. One participant believed the use of mHealth apps would help doctors to “...track patient progress at any time” (nonuser 15). Others perceived mHealth apps as a form of a medical emergency solution that will alert health authorities about an emergency by the simple tap of a button. One other highly mentioned feature was *reminders*, a feature that most current mHealth users believe has made their mHealth solutions become their *“*companions.” Both users and nonusers of mHealth solutions believe the availability of reminders in mHealth solutions plays an enabling role in them taking such actions as taking medication, making medical appointments, and keeping active:

...My watch tells me to move or stand up if I’ve been sitting or not moved for an hour.

User 31

Other highlighted features include “...quick tips” (nonuser 13), “quick communication with your medical team” (user 20), “...rehabilitation tutorial videos and coaching support” (nonuser 16), “...track diet and physical activity” (user 29), and visualization of reports for “pain levels, medicine intake, heart rate, and stress levels” (nonuser 18).

Concerning met and unmet expectations for mHealth solutions, most users pointed to data accuracy as a significant unmet expectation. They mentioned inaccuracies in measurements, such as blood pressure and daily step count. Others shared their frustrations with being swamped with in-application ads (for monetizing by app developers), software glitches, and subscription-based features. One participant noted that the amount of data required to be entered was daunting:

...Too many options and things to fill in, it makes me panic.

User P11

Although mHealth solutions are ubiquitous and allow monitoring one’s health and activities in different ways, it is worth noting that mHealth solutions are aid and, therefore, cannot satisfy all conditions. One such concern was raised by a user about the unsuitability of their mHealth solution for tracking their pain:

...My chronic pain is on my back, so it’s not trackable by my activity tracker.

User 26

### Reliability

We further sought to understand the expectations of mHealth nonusers and the realizations of mHealth users toward the reliability of mHealth solutions. Participants were quizzed about the degree to which the solution will continuously operate properly and the validity of its information sources. Unsurprisingly, a vast majority of concerns about reliability came from nonusers. To this end, nonusers expressed high expectations of mHealth solutions to be reliable. To some, the reliability of mHealth solutions to deliver what is expected of them was crucial for adopting and using mHealth solutions in the first place. As shown in Figure 2, mHealth users found their mHealth solutions generally reliable, and nonusers also expected similar reliability. The most significant discrepancy we identified was in source credibility (ie, how well the solutions are backed by science or designed in conjunction with medical experts). Here, 18 (58%) of the mHealth users were not concerned about source credibility, while 29 (94%) nonusers were concerned about it (mean 3.51, SD 1.89, vs mean 6.19, SD 0.90; *P*<.01).

The reliability of mHealth solutions is so important that if they are not reliable, then “...there would be no good in using them” (nonuser 6). Some highlighted that mHealth solutions have human health at stake and that there cannot be room for error. Put bluntly, 1 participant highlighted the need for credibility and reliability of mHealth solutions because “...they are dealing with health which is important and delicate” (nonuser 5) and, as a result, “...there can be no room for mistakes to be made” (nonuser 15). In this regard, some participants noted the potential negative consequences of mHealth solutions providing unreliable data, a situation that can lead to incorrect diagnosis and pain management routines, a sentiment shared by other participants.

Taking a more realistic approach, 1 nonuser cautioned against having too high expectations of technology, stating, “technology is fallible” (nonuser 17). The inalienable fact of human error was also mentioned, with a participant noting that human error can contribute to potential reliability issues:

I don’t expect it to be 100% reliable because it will be using information that I give it. I may sometimes make an error or overestimate the amount of pain I am experiencing.

Nonuser 28

### Usefulness

Generally, participants expressed a positive complementary role mHealth solutions could play in understanding their pain, accessing and discussing issues with health professionals, and allowing health professionals to monitor data about such pain. To the ordinary user, the ability to visualize their data to gain better clarity to better explain things to health care professionals is of importance to them. Although most participants agreed that mHealth solutions could not replace medical doctors, nor can suggestions in mHealth apps surpass those of medical doctors, they can play a complementary role in assisting users better understand their pain. As such, participants noted that monitoring one’s chronic pain would be much easier and more effective for both patients and health care professionals as the mHealth solutions would have vital information for doctors to view and monitor the patient’s condition on an ongoing basis, which would allow for more effective treatment over time.

To analyze the answers of participants to each question quantitatively in more detail, as demonstrated in Figure 3, 24 (77%) of the mHealth users (vs n=27, 87%, of nonusers) found mHealth solutions helpful in tracking or managing their chronic pain (mean 5.09, SD 1.30, vs mean 5.74, SD 1.12; *P*=.02). In addition, 15 (48%) of the mHealth users (vs n=19, 61%, of nonusers) declared that mHealth solutions save their time, while 16 (52%) of the mHealth users (vs n=23, 74%, of nonusers) expressed that using mHealth solutions has reduced their overall concern about their chronic pain (mean 4.09, SD 1.90, vs mean 5.12, SD 1.47; *P*=.01). Only 2 (6%) of the mHealth users (vs n=5, 16%, of nonusers) disagreed that using mHealth solutions has increased their control over their daily activity. In addition, 18 (58%) of the mHealth users (vs n=24, 77%, of nonusers) found mHealth solutions helpful to maintain their chronic pain, while 12 (39%) of the mHealth users (vs n=22, 71%, of nonusers) declared that using mHealth solutions has made interacting with health or social care professionals easier (mean 3.64, SD 2.15, vs mean 5.06, SD 1.65; *P*=.01), and 23 (74%) of the mHealth users (vs n=25, 81%, of nonusers) found following medical advice, treatment guidelines, or any potential exercise routine easier than using mHealth solutions. Only 9 (29%) of the mHealth users (vs n=21, 68%, of nonusers) declared that their doctor uses mHealth solutions’ data in-office visits (mean 2.93, SD 2.24, vs mean 4.93, SD 1.56; *P*<.01).

Users of mHealth solutions shared an overarching sentiment of mHealth solutions, aiding them in following their routines, taking medicines, monitoring weight loss goals, exercising, managing food intake, monitoring pain levels, etc. One user noted:

It’s been useful to track the days I’m feeling worse or better.

User 15

The use of mHealth solutions has also enhanced communication between patients and doctors by making data available for medical staff to analyze, a fundamental expectation of the nonusers. As one user put:

[mHealth solutions have been] useful in presenting data to my practitioner.

User 18

Not all participants had such high expectations for mHealth solutions as mHealth solutions may not suit all conditions. According to 1 participant, using mHealth solutions would not offer any benefit to them:

In my case, such a service would not be of much use at all.

Nonuser 3

### Usage Satisfaction and Other Expectations and Impressions

To investigate the general satisfaction of using mHealth solutions as well as the expected satisfaction of nonusers, participants were quizzed with 3 questions. As depicted in Figure 4, 26 (84%) of the mHealth users (vs n=25, 81%, of nonusers) said they would recommend mHealth solutions to others suffering from chronic pain. In addition, 29 (94%) of the mHealth users said they would continue to use mHealth solutions in the future, while 26 (84%) of the nonusers said they would use mHealth solutions in the future (mean 6.03, SD 1.16, vs mean 5.19, SD 1.66; *P*=.01), and 23 (74%) of the users found mHealth solutions fun, while only 20 (65%) of the nonusers expected mHealth solutions to be fun. On average, 26 (84%) of the mHealth users (vs n=24, 76%, of nonusers) were satisfied with mHealth solutions.

### Privacy and Data Management

Participants were asked about their concerns for privacy and the importance of owning and sharing their data. We noticed that real-world events, such as data breaches, that have occurred worldwide influenced the participants’ position on their willingness to donate their data. One nonuser noted:

There have been numerous cases where private data were mistreated.

Nonuser 6

In addition to the fear of data abuse or misuse, participants also expressed concern for the lack of knowledge on how their data are being protected or even sometimes used for purposes unknown to the data donor:

I have concerns about my private information being used for other purposes.

Nonuser 21

Qualitative and quantitative results showed that mHealth nonusers are more concerned about their privacy compared to users. As shown in Figure 5, 26 (84%) of the nonusers expressed concern about sharing their data with, for example, third parties, while only about half (n=15, 48%) of the mHealth users (mean 4.00, SD 1.86, vs mean 5.77, SD 1.25; *P*<.01) were concerned.

### Willingness to Donate Personal Data

We went a step further to inquire from participants what types of personal data they would be willing to give to gain additional benefit from the use of their mHealth solutions. Our goal was to understand how willing people are to donate sensitive personal data. Although most did cite privacy concerns for their unwillingness to donate their data, some were not worried about the data they already share on, for example, social media:

Most data that I would be likely to share is also willingly shared by me on social media.

Nonuser 10

Any additional benefits from donating more of their data were generally received negatively. mHealth users, in particular, had serious reservations about their sensitive data, such as fingerprints, mentioning the fear of identity theft as a major concern:

I don’t want to share my fingerprints or national identification because someone can take credit for me or steal my identity.

User 4

Iris, fingerprints and location data seem way too private for a medical app to have.

User 20

However, some participants were willing to donate such data if they could be guaranteed anonymity:

I do not want anyone else having personally identifiable information; they can have anonymized info or info that will not identify me, though.

User 21

### Ownership and Control of Personal Data

Most mHealth users believed that they and the mHealth solution provider deserve access to their data. As such, 17 (55%) of them were unconcerned that mHealth solution manufacturers have access to their data. On the contrary, only 6 (19%) of the nonusers (mean 3.48, SD 2.14, vs mean 4.87, SD 1.40; *P*<.01) were unconcerned. mHealth users believed that granting the mHealth solution provider access to their data will enable the provider to “show me better-organized results for example” (user 16). Another participant put it in more precise terms:

Surely I should have some degree of control as well as the provider for data analysis purposes and data management.

User 20

However, concerning control of the data, mHealth users believed they reserve the right to control their data as the data belong to them:

It’s my data so I should be in charge of it.

User 31

It is my data, so I should have full ownership and control.

Nonuser 25

Concerning the potential for misuse of people’s data, 1 participant made a plea for the introduction of a third noncommercial party to manage the access and use of personal data to curtain the misuse of such data. The participant noted:

Data can be misused by commercial enterprises, some level of neutrality (and non-commerciality) might mitigate against this.

User 18

Concerning the handling of personal data, 18 (58%) of the mHealth users believed that mHealth solution providers have safely handled their collected data. In comparison, 20 (65%) of the nonusers expected safe handling of their data.

### Future of Personal Data

Lastly, we sought to elicit the participants’ opinions on how they envision the future of their data to be in terms of data management. Most nonusers said that they would like to manage their data, but failed to state *how* that could be achieved. However, 1 nonuser was adamant that a neutral third party was the way to go in this light:

I think that data management should be done by a neutral third party specialized in the field.

Nonuser 10

One interesting observation was a call by 1 participant for their data to be linked to an insurance provider:

I would prefer if my health data was specifically linked to my health insurance provider.

Nonuser 9

Although most participants were adamant about a future where they have control over their data, 1 participant made a somewhat dystopian claim, suggesting an end to personal data ownership and control:

I imagine soon we won’t own any data at all. It will all be taken from us.

User 18

### Reasons for Not Using mHealth Solutions in Nonusers’ Viewpoint

We sought to understand from mHealth nonusers their reasons for not using mHealth solutions. A lack of knowledge about the existence and potential benefits of using mHealth solutions was a significant sentiment shared by the majority of the participants, with most voicing concern about neither knowing about mHealth solutions at all nor knowing about their potential benefits. One user noted:

I am not familiar with this product/service and the benefits it can bring to my pain.

Nonuser 30

Some participants mentioned that there was simply no need to track their pain. In contrast, others believed they do not use mHealth solutions because it has not been recommended to them by medical health professionals. Those, however, who have known about mHealth solutions but do not use them highlighted financial cost as a major barrier as it “...can be a bit expensive” (nonuser 11) and that most mHealth solutions “...come with costs and ads” (nonuser 21). Some participants, however, were confident they do not need mHealth solutions:

I don’t think I need it. I can manage my pain without it.

Nonuser 28

## Discussion

### Principal Findings

As wearable devices, such as Fitbit, Oura Ring (Ōura Health Oy, Finland), and Apple iWatch, are increasingly being highlighted in the media as solutions to improve people’s well-being, mHealth represents a class of technologies that are set to proliferate in the near future. Yet, this domain is still a young and unregulated one, and only a fraction of the mHealth apps in the digital app stores online have undergone a rigorous evaluation. Thus, their real impact remains unknown. Our study set out to explore mHealth expectations of mHealth nonusers and experiences of mHealth users for a specific user group, people with chronic pain.

Our sample of mHealth users is naturally self-selecting, as they have chosen to adopt mHealth of their own volition. Thus, it is not easy to comprehensively judge whether, for example, the expectations people have for mHealth would change after they adopt such solutions or whether the people who use mHealth devices have done so due to their earlier expectations. Yet, we argue that the quantitative and qualitative data we present in this paper act as a solid conversation starter to understand the contrast between these 2 groups of people (mHealth users and mHealth nonusers).

### The Future Role of mHealth Devices

The most mentioned feature overall by the 62 participants was *reminders*, which appeals to both users and nonusers as they expressed the need to rely on reminders to “take medicine” or even “take a break,” much in line with Zhou et al [35] and Siegler et al [36]. Participants expressed a desire for mHealth solutions to become part of their life, as some referred to it as a *companion* [79]. Companion technologies fill the gap between the broad functionality of technical systems and human users’ individual needs. They aim to appear as “companions” to their users. They enable the construction of smart, adaptive, flexible, and cooperative technical systems by applying and combining methods from various research areas. They serve as cooperative agents assisting in specific tasks or even give companionship to humans in a more general sense [80,81]. Variant companions in the HCI field have been developed, such as Artificial Commensal Companions to provide social interactions during food consumption [82], the Prayer Companion that can be used as a resource for the spiritual activity of a group of cloistered nuns [83], and the Flippo that is a social wearable creature prototype and is meant to support people to take breaks away from their desks and move [84]. To this end, as some participants with CLBP expressed that their chronic pain is not trackable with their activity trackers, mHealth solutions could be developed as companion solutions to manage each specific chronic pain, such as CLBP, separately and more professionally. In contrast, most of our participants expressed optimism about mHealth solutions being critical to helping them become independent in managing their pain (in line with Pfeifer et al [14], Yang et al [40], and Amorim et al [85]) by providing interventions just in time, as also discussed by Künzler [86].

Participants further expressed a high expectation of mHealth solutions to be reliable in delivering accurate results and performance. Reliable data present an opportunity for individuals to communicate with their health care providers (eg, doctors and therapists) regarding their pain. By presenting data from these mHealth solutions, participants believed their health care providers would assist them in managing their pain better. At this point, the question is then about finding functional solutions that medical experts would be willing to adopt on a broader scale. One obstacle in using these devices more broadly is data management, with all types of concerns for privacy or misuse, such as sharing with a third party, selling to a third party, and having access of the wrong personnel to mHealth [35,87].

### It Is Always About the Data: Insights Into the Perceived Privacy of mHealth Solutions

People expect information to flow in a certain way in a given situation. When it does not, privacy concerns may arise. The benchmark of privacy is contextual integrity (ie, in any condition, a complaint that privacy has been violated is sound if 1 or other types of informational norms have been transgressed [88]). The sensitive nature of personal data that such mHealth apps access poses a problem to data privacy [30].

Studies show that mHealth apps on Google Play Store contain codes that could potentially collect user data and transmit them to their traffic [30] or share the users’ information with a third party [89]. mHealth solutions developers routinely and legally share user data with third parties, often in exchange for services that enhance the user experience or monetize the app [90]. The participants were also aware of data privacy breaches and expressed their concerns.

However, little transparency exists in third-party data sharing, and mHealth solutions routinely fail to provide any assurances despite collecting and transmitting multiple forms of personal data [91,92].

Participants in our study considered themselves moderately to highly aware of their data privacy rights, and their responses echoed sentiments of not only being aware of but also contributing to their information privacy. Interestingly, although individuals tend to declare their concern about privacy, their actions often belie such claims [77,93]. Individuals passively trade their personal information in exchange for access to use various apps for free (sign up and use for free); they accept terms and conditions without reading them and willingly share sensitive information on social media—a behavior exhibited by our study participants, as well.

We found that our participants, on the one hand, stated that they were concerned about privacy. Yet, on the other hand, they demonstrated diversity in views about the future of management. For example, when it comes to what data to donate, they expressed that they do not trust the platforms with that information. However, in responding to their thoughts on the future management of their health data, we see a contrasting view that they would prefer that their health data were specifically linked to their health insurance provider. In a related paper, Solove [94] stated that people are prone to shouting, “That violates my privacy,” while lacking clarity on what privacy actually means [77,94]. As such, we find that privacy discussions mostly appeal to people’s fears and anxieties, in line with Alorwu et al [87] and Solove [94]. However, we were excited about what types of personal data our respondents were willing to donate in exchange for additional benefits. We are confident that the results demonstrate *healthy carefulness.* Passwords and pins were the main traditional methods that some apps chose to secure themselves. With the increasing vulnerabilities of both passowrds and pins due to hacker attacks, biometrics is a good alternative for mobile apps to reduce cyber security threats. In some countries, people need to enter their health care system using their bank account number, and it is worth noting that the banking and finance sector has nearly universally embraced biometric security systems as the primary way to secure access to their apps and services. However, our participants were reluctant to donate their debit or credit card numbers, and their biometrics data included iris patterns and fingerprints. In line with previous research (eg, Presthus and Sørum [77]), the top 3 personal data participants were willing to donate were their first name, last name, and email address.

### Managing Chronic Pain With mHealth

A variety of life events cause chronic pain: injuries, surgery, illnesses, or age. Managing chronic pain is not an easy process, as it is often long term and requires a lot of patience. The degree of pain experienced by people also differs. To this end, mHealth solutions that aim to help people manage their pain should be able to do so without imposing any extra burden and complicating things further. The participants mainly stated their experiences and expectations in health management; the reason might be the generality of the data that mHealth solutions track and collect. To be more related to managing pain, the mHealth solutions need to have more specific features to measure different items. Hereupon, their development requires a diverse set of expertise, including software programmers, behavioral scientists, graphical designers, and medical experts, such as doctors and physiotherapists. It also requires end-user feedback about the solutions themself so that the vendors can match actual users’ real-life needs [38,39]. However, medical experts are still reluctant to use mHealth solutions as part of their treatment despite the perceived potential, due to insufficient evidence of their benefits [5].

Further, from the medical standpoint, the cost of managing chronic pain can be high, especially for those with a low socioeconomic status to begin with. The cost of higher-end mHealth solutions could be simply too much. Our results also indicate that most mHealth users began using mHealth solutions by their own initiative. Only a handful did so through recommendations from friends, medical doctors, or therapists. As our results show, mHealth users perceive value in their use, so it is fair to speculate that perhaps their wider adoption could be helpful more broadly for others with pain, too. Could doctor-recommended mHealth solutions offer greater benefits to users?

### Implications for the Future of mHealth

Based on our study, we bring forward specific implications. First, we believe there is a potential missed opportunity by mHealth manufacturers by not specifically aiming to make communications with clinicians easier. The nonusers among our participants were expecting the mHealth solutions to facilitate this, yet this was not the case according to the users.

Second, nonusers were more concerned than users about the mHealth solutions having access to personal data and the solution providers sharing those data with third parties. To this end, transparent and ethical data management, and communicating all this to potential new users of mHealth solutions, is critical to driving further mHealth adoption.

Finally, nonusers were significantly more concerned about the source credibility of mHealth solutions. This could be potentially an unfortunate misunderstanding, as the purpose of mHealth is certainly not just to provide accurate medical assistance or even science-backed aid. Many mHealth solutions are simply used to track users’ activity or even provide information content based on users’ preferences. Again, this offers an excellent way to build credibility for mHealth providers but also a communication opportunity: not all solutions have to be medical grade to fulfill their promise to the consumers.

### Limitations

We admit some limitations of our study. Our sample is not representative of mHealth users as a population. However, we argue it is sufficient for discussing the emerging differences. A larger sample is needed for the increased generalizability of our results. Further, sourcing participants from multiple sources would be a more optimal sampling strategy. To this end, Prolific has been shown to yield valid data for questionnaire studies in HCI and other fields.

We acknowledge that using 2 different samples for *expectations* and *actual experience* is less optimal than, for example, exploring the pre- and postadoptance behavior of 1 sample. Our study could potentially be impacted and moderated by social factors, such as demographics, education, income, or marital status. If we had used the same samples in a longitudinal study, the results would potentially differ. However, for our purposes of a questionnaire study, using 2 samples drawn from a reliable human subject pool online, we argue, is adequate.

Our results indicate a trend toward people who do not use mHealth solutions, being more concerned about their privacy than those who use mHealth solutions. However, we cannot know whether people who are less concerned about privacy issues are those who adopt and use mHealth solutions or whether the use of mHealth solutions makes people worry less about their privacy. This presents a promising future research opportunity.

### Future Work

We plan to extend this work by conducting a longitudinal study by letting users elicit their expectations before using an mHealth solution. We will then contrast their felt-experience against their expectations. This will help improve the understanding of how mHealth could be critical to managing chronic pain in the future. Another exciting opportunity is a thorough cross-validation of results obtained through crowdsourced marketplaces (eg, Prolific in our case) and larger-scale questionnaire studies online. Finally, further studies should be conducted with medical professionals to acquire their expert feedback on how mHealth solutions could be helpful to the self-management of conditions such as chronic pain.

### Conclusion

In this paper, we investigated how mHealth is currently used in the context of chronic pain and the expectations of mHealth nonusers for mHealth as a future chronic pain management tool. We conducted 2 studies, an initial study to identify people who use mHealth and a follow-up study to elicit insights into mHealth expectations and experiences between mHealth users and nonusers. Our analysis reveals contrasts between mHealth use expectations and actual usage experiences and highlights privacy concerns regarding mHealth solutions.

## Appendix

Multimedia Appendix 1 Questionnaire for people with chronic pain to investigate how many use mobile health (mHealth).

Multimedia Appendix 2 Questionnaire for people with chronic pain who use mobile health (mHealth).

Multimedia Appendix 3 Questionnaire for people with chronic pain who do not use mobile health (mHealth).

## Abbreviations

CLBP: chronic low back pain
EDT: expectation disinformation theory
HCI: human-computer interaction
Health-ITUES: Health Information Technology Usability Evaluation Scale
IoT: internet of things
mHealth: mobile health
MWW: Mann-Whitney-Wilcoxon
SUTAQ: Service User Technology Acceptability Questionnaire
TUQ: Telehealth Usability Questionnaire
UI: user interface
WHO: World Health Organization
