# Supplementary material for: Contrasting the Expectations and Experiences Related to Mobile Health Use for Chronic Pain: Questionnaire Study
Source: JMIR Hum Factors. 2022 Sep 6;9(3):e38265. doi: 10.2196/38265 (PMC9490547; doi:10.2196/38265)
Supplement: Multimedia Appendix 1 [file humanfactors_v9i3e38265_app1.pdf]

Chronic Pain Questionnaire

The purpose of this study is to learn about how and with what tools people with chronic pain manage their pain.

This questionnaire will take approximately 5-10 minutes of your time and your responses will remain fully anonymous.

By continuing to take this questionnaire, you consent to your answers being used in an academic research and hopefully to the benefit of other people suffering from various types of chronic pain.

This is a pre-questionnaire we use to collect information about individuals with chronic pain and how they use m-Health technology to track and manage their conditions. We will ask responders to participate in a follow-up questionnaire which will inquire about the use of m-Health technology in more detail.

We really appreciate your input!

* Required

1. Give my consent *

Mark only one oval.

◯ By continuing with this questionnaire, I grant consent to the researchers to collect data about me for the purposes of this research. I understand the privileges I have towards my own data according to the GDPR legislation.

2. Your unique Prolific ID. (DO NOT EDIT) *

____

General information about your chronic pain and tracking methods

README FIRST: What is m-Health?

We are interested in mobile health (m-Health). M-Health is a term used in practicing medicine to advance public health with the support of mobile devices. M-Health is most commonly associated with mobile communication devices, such as mobile phones, tablet computers, personal digital assistants (PDAs), and wearable devices such as smartwatches and activity trackers, bracelets, and other forms of wearable technology. M-Health devices can be used for e.g., health services, information dissemination, and data collection.

In other words, m-Health refers to the concept of mobile self-care — consumer technologies like wearables, smartphones and tablet apps that enable consumers to capture their own health data without a clinician's assistance or interpretation.

3. What chronic pain do you have? (you can select multiple options) *

Check all that apply.

- [ ] Primary pain
- [ ] Cancer pain
- [ ] Postsurgical or posttraumatic pain
- [ ] Neuropathic pain
- [ ] Headache and orofacial pain
- [ ] Visceral pain
- [ ] Musculoskeletal: Arthritis
- [ ] Musculoskeletal: Back pain
- [ ] Musculoskeletal: Joint pain
- [ ] Other: ____________________

4. How many years have you experienced chronic pain? *

(Supports decimal numbers, e.g. for six months you can write 0.5)

____

5. How do you manage your pain, in general? *

Write as much as you want. There are no wrong answers.

____

____

____

____

____

REMINDER: What is m-Health?

We are interested in mobile health (m-Health). M-Health is a term used in practicing medicine and advancing public health supported through mobile devices. M-Health is most commonly used about using mobile communication devices, such as mobile phones, tablet computers, personal digital assistants (PDAs), and wearable devices such as smartwatches and activity trackers, bracelets, and other form of wearable technology. M-Health devices can be used for e.g., health services, information collection, and data collection.

In other words, m-Health refers to the concept of mobile self-care — consumer technologies like wearables, smartphones and tablet apps that enable consumers to capture their own health data without a clinician's assistance or interpretation.

6. Do you use any m-Health solutions (as described in this questionnaire) to track or manage your pain? *

Mark only one oval.

- ( ) Yes
- ( ) No

7. If you answered yes to the previous item, please let us know which m-Health solutions you use, and how?

____
____
____
____
____

Confirmation message

Our study methodology involves re-visiting and reformatting these questions to better understand the use of m-Health interventions. We may invite you to a follow-up study in the near future.

This content is neither created nor endorsed by Google.

Google Forms
