# Supplementary material for: Contrasting the Expectations and Experiences Related to Mobile Health Use for Chronic Pain: Questionnaire Study
Source: JMIR Hum Factors. 2022 Sep 6;9(3):e38265. doi: 10.2196/38265 (PMC9490547; doi:10.2196/38265)
Supplement: Multimedia Appendix 2 [file humanfactors_v9i3e38265_app2.pdf]

Questionnaire for M-Health Users

In an earlier study by us, you indicated to use m-Health solutions. This time, we are hoping to learn more about how you use m-health solutions, especially concerning chronic pain. This questionnaire will take approximately 30-40 minutes of your time and your responses will remain fully anonymous.

By continuing to take this questionnaire, you consent to your answers being used in an academic research and hopefully to the benefit of other people to use m-health solutions to manage their chronic pain.

We really appreciate your input, and we will pay extra bonuses for particularly insightful answers!

* Required

1. Consent *

Mark only one oval.

( ) Give my consent and continue taking the questionnaire

README FIRST: What is m-health?

We are interested in mobile health (m-Health). M-Health is a term used in practicing medicine to advance public health with the support of mobile devices. M-Health is most commonly associated with mobile communication devices, such as mobile phones, tablet computers, personal digital assistants (PDAs), and wearable devices such as smartwatches and activity trackers, bracelets, and other forms of wearable technology. M-Health devices can be used for e.g., health services, information dissemination, and data collection.

In other words, m-Health refers to the concept of mobile self-care — consumer technologies like wearables, smartphones and tablet apps that enable consumers to capture their own health data without a clinician's assistance or interpretation.

2. A: What m-Health solution(s) do you use? *

3. B: More specifically, how do you use, if at all, these solutions in relation to chronic pain? *

E.g. do you track, monitor, manage your chronic pain with such devices? Or do you use them to support pain management in some other ways?

4. C: How long have you been using m-Health solution(s) and how often do you use them? *

Please try to provide accurate answers: E.g. several times per day, daily, weekly, etc.

5. D: Did you start to use m-Health solution(s) by your own choice or because someone else, e.g. a medical expert, has advised you to do so? *

If recommended by someone, who? Why? Try to be as accurate as you can.

Ease of use

Here, we are interested in "the degree to which the sensing solution will require little mental effort to use". Please consider the m-Health solutions you personally use, and answer the following items:

6. How easy did you find the use of m-Health solution(s) that you use? *

Mark only one oval.

	1	2	3	4	5	6	7	
Not at all easy	⬭	⬭	⬭	⬭	⬭	⬭	⬭	Extremely easy

7. How easy did you find learning to use m-Health solution(s) that you use? *

Mark only one oval.

	1	2	3	4	5	6	7	
Not at all easy	⬭	⬭	⬭	⬭	⬭	⬭	⬭	Extremely easy

8. How easy was it for you to become skillful at using m-Health solution(s) that you use? *

Mark only one oval.

	1	2	3	4	5	6	7	
Not at all easy	⬭	⬭	⬭	⬭	⬭	⬭	⬭	Extremely easy

9. Please elaborate on your choices in the previous questions. Anything that comes to mind is welcome, feel free to simply discuss briefly issues around ease of use. *

10. Please describe any problems or issues that you had to tackle or somehow address in your m-Health solutions concerning ease of use. *

Functionality

Here, we want you to consider "the degree to which the solution will have the capability, functions, or features needed to accomplish its tasks."

11. Please describe the features and functions that you expect from m-Health solutions related to your chronic pain? *

12. How well do the m-Health solution(s) you use fulfill your expectations (features, functions, etc.) *

Mark only one oval.

	1	2	3	4	5	6	7	
Not at all fulfilling	◯	◯	◯	◯	◯	◯	◯	Extremely fulfilling

13. Please elaborate on your answer above: Why do the m-Health solution(s) that you use meet or do not meet your expectations? *

Reliability

Now consider "the degree to which the solution will continually operate properly, or will operate in a consistent, flawless manner."

14. How reliable do you find m-Health solution(s) that you use in general? *

Mark only one oval.

	1	2	3	4	5	6	7	
Not at all reliable	⬭	⬭	⬭	⬭	⬭	⬭	⬭	Extremely reliable

15. How concerned are you about the source credibility of m-Health solutions? *

By "source credibility" we mean that the solution is based on any expert information and algorithms.

Mark only one oval.

	1	2	3	4	5	6	7	
Not at all concerned	⬭	⬭	⬭	⬭	⬭	⬭	⬭	Extremely concerned

16. Please describe any reliability problems or issues in your m-Health solution(s) that you had to tackle or somehow address? *

Usefulness

Now consider "the degree to which the user trusts that the solution is beneficial for its purpose."

17. How useful do you find m-Health solutions to track or manage your chronic pain in general? *

Mark only one oval.

	1	2	3	4	5	6	7	
Not at all useful	◯	◯	◯	◯	◯	◯	◯	Extremely useful

18. How much does your doctor use information from your m-Health solution(s) in office visits? *

Mark only one oval.

	1	2	3	4	5	6	7	
Not at all much	◯	◯	◯	◯	◯	◯	◯	Extremely much

19. How much easier is it to follow medical advice, treatment guidelines, or any potential exercise routine you are following since starting to use m-Health solutions? *

Mark only one oval.

	1	2	3	4	5	6	7	
Not at all much	◯	◯	◯	◯	◯	◯	◯	Extremely much

20. Are m-Health solutions helpful in reducing your overall concern about your chronic pain? *

Mark only one oval.

	1	2	3	4	5	6	7	
Not at all helpful	◯	◯	◯	◯	◯	◯	◯	Extremely helpful

21. How much do m-Health solutions help you in maintaining your chronic pain? *

Mark only one oval.

	1	2	3	4	5	6	7	
Not at all much	◯	◯	◯	◯	◯	◯	◯	Extremely much

22. How much time, if any, do you save because of using m-Health solutions? *

For instance, do not go to clinique and send your data to your doctor

Mark only one oval.

	1	2	3	4	5	6	7	
Not at all much	◯	◯	◯	◯	◯	◯	◯	Extremely much

23. How much easier have m-Health solutions made it to interact with health or social care professionals? *

Mark only one oval.

	1	2	3	4	5	6	7	
Not at all much	◯	◯	◯	◯	◯	◯	◯	Extremely much

24. How much more control do m-Health solutions give you over the activities in your life? *

Mark only one oval.

	1	2	3	4	5	6	7	
Not at all	◯	◯	◯	◯	◯	◯	◯	Extremely

25. Please describe any other aspects in which you think that m-Health solutions have been useful, in the context of your chronic pain. *

____
____
____
____
____

Other expectations and impressions

26. How much are you planning to use m-Health solutions in the future? *

Mark only one oval.

	1	2	3	4	5	6	7	
Not at all much	◯	◯	◯	◯	◯	◯	◯	Extremely much

27. How fun do you find m-Health solutions to be? *

Mark only one oval.

	1	2	3	4	5	6	7	
Not at all fun	◯	◯	◯	◯	◯	◯	◯	Extremely fun

28. How much would you recommend m-Health solutions to other people who are in a similar situation to you (they also have chronic pain)? *

Mark only one oval.

	1	2	3	4	5	6	7	
Not at all much	◯	◯	◯	◯	◯	◯	◯	Extremely much

Privacy etc.

29. How much do you think m-Health solutions invade your privacy? *

Mark only one oval.

	1	2	3	4	5	6	7	
Not at all much	◯	◯	◯	◯	◯	◯	◯	Extremely much

30. In your estimate, how safe are your data collected through your m-Health solutions treated? *

Mark only one oval.

	1	2	3	4	5	6	7	
Not at all safe	◯	◯	◯	◯	◯	◯	◯	Extremely safe

31. How concerned are you about your m-Health solution manufacturer having access to your personal data collected via the m-Health solution? *

Mark only one oval.

	1	2	3	4	5	6	7	
Not at all concerned	◯	◯	◯	◯	◯	◯	◯	Extremely concerned

32. How concerned are you about your personal data being shared with, e.g., third parties without your permission? *

Mark only one oval.

	1	2	3	4	5	6	7	
Not at all concerned	⬭	⬭	⬭	⬭	⬭	⬭	⬭	Extremely concerned

33. How likely are you to donate personal data of different types in order to gain some additional benefits from the use of the m-Health solution, e.g. an additional feature, a PRO version of an app, discounts, etc? *

Mark only one oval per row.

	1- Not at all likely	2	3	4 - Neutral	5	6	7 - Extremely likely
**First name**	◯	◯	◯	◯	◯	◯	◯
**Surname**	◯	◯	◯	◯	◯	◯	◯
**Email address**	◯	◯	◯	◯	◯	◯	◯
**Phone number**	◯	◯	◯	◯	◯	◯	◯
**Residential address**	◯	◯	◯	◯	◯	◯	◯
**Iris patterns**	◯	◯	◯	◯	◯	◯	◯
**Fingerprints**	◯	◯	◯	◯	◯	◯	◯
**Birth dates and national identification number**	◯	◯	◯	◯	◯	◯	◯
**Debit/Credit card number**	◯	◯	◯	◯	◯	◯	◯
**Location data**	◯	◯	◯	◯	◯	◯	◯

34. Please tell us your reasons for answering the way you did in the previous question? *

35. Considering the data you produce with the m-Health solution you use, who do you think should be in control of your data. *

Mark only one oval.

- Only me
- Myself and the m-health solution provider
- The m-health solution provider
- A neutral third-party e.g. a data intermediary established just for data management purposes
- It does not matter

36. Please tell us your reasons for answering the way you did in the previous question? *

37. What are your thoughts on the future management of your personal health data? *

Feel free to express your thoughts freely. E.g. Who should have access to it? How should it be shared? Where should it be stored? Who should pay for the cost of storage and protection? There are no right or wrong answers.

This content is neither created nor endorsed by Google.

Google Forms
