# Supplementary material for: Contrasting the Expectations and Experiences Related to Mobile Health Use for Chronic Pain: Questionnaire Study
Source: JMIR Hum Factors. 2022 Sep 6;9(3):e38265. doi: 10.2196/38265 (PMC9490547; doi:10.2196/38265)
Supplement: Multimedia Appendix 3 [file humanfactors_v9i3e38265_app3.pdf]

Questionnaire for M-Health Non-Users

You took part earlier in a short questionnaire about m-Health and indicated that you do not use any such solutions for your pain management needs. The purpose of this study is to find the reasons for not using m-Health solutions.

This questionnaire will take approximately 30-40 minutes of your time and your responses will remain fully anonymous.

By continuing to take this questionnaire, you consent to your answers being used in an academic research and hopefully helping people to improve using m-health solutions to manage their chronic pain.

We really appreciate your input!

* Required

1. Give my consent *

Mark only one oval.

◯ Give my consnet and continue taking the questionnaire

README FIRST: What is m-Health?

We are interested in mobile health (m-Health). M-Health is a term used in practicing medicine to advance public health with the support of mobile devices. M-Health is most commonly associated with mobile communication devices, such as mobile phones, tablet computers, personal digital assistants (PDAs), and wearable devices such as smartwatches and activity trackers, bracelets, and other forms of wearable technology. M-Health devices can be used for e.g., health services, information dissemination, and data collection.
In other words, m-Health refers to the concept of mobile self-care — consumer technologies like wearables, smartphones and tablet apps that enable consumers to capture their own health data without a clinician's assistance or interpretation.

If you have used any m-Health solutions ever, please answer the next four questions.

If you have not used just write "NO" in the next four questions.

2. A: Have you ever used any m-Health solution(s) to track or manage your chronic pain? What m-Health solutions? *

3. B: More specifically, how have you used, if at all, these solutions in relation to chronic pain? *

E.g. have you tracked, monitored, managed your chronic pain with such devices? Or have you used them to support pain management in some other ways?

4. C: How long and how often have you used m-Health solution(s)? *

Please try to provide accurate answers: E.g. several times per day, daily, weekly, etc.

5. D: Did you start to use m-Health solution(s) by your own choice or because someone else, e.g. a medical expert, has advised you to do so? *

If recommended by someone, who? Why? Try to be as accurate as you can.

If you have not used any m-Health solutions ever, please answer the next question.

6. If you have not used any m-Health solutions ever, please answer this question: why have you not used m-Health solutions to track or manage your chronic pain ever? *

Explain all the reasons that lead to not using m-health solutions so far.

Ease of use

Here, we want you to consider all the expectations of "the degree to which the sensing solution will require little mental effort to use".

7. How easy would you expect the use of m-Health solution(s) to be? *

Mark only one oval.

	1	2	3	4	5	6	7	
Not at all easy	◯	◯	◯	◯	◯	◯	◯	Extremely easy

8. How easy would you expect learning to use m-Health solution(s) to be? *

Mark only one oval.

	1	2	3	4	5	6	7	
Not at all easy	⬭	⬭	⬭	⬭	⬭	⬭	⬭	Extremely easy

9. How easy would you expect becoming skillful at using m-Health solution(s) to be? *

Mark only one oval.

	1	2	3	4	5	6	7	
Not at all easy	⬭	⬭	⬭	⬭	⬭	⬭	⬭	Extremely easy

10. Please elaborate on your choices in the previous questions. Anything that comes to mind is welcome, feel free to simply discuss briefly all the expectations that you have about easiness. *

11. If you have previously used any m-Health solutions, please describe any problems or issues that you had which might affected your reason to stop using them. *

Functionality

Here, we want you to consider all the expectations of "the degree to which the solution will have the capability, functions, or features needed to accomplish its tasks."

12. Please describe the features and functions that you expect from m-Health solutions related to your chronic pain? *

13. If you have previously used any m-Health solutions, please describe how the solutions you have used fullfilled your expectations. *

14. Please elaborate on your answer above: Why did the m-Health solution(s) that you used meet or did not meet your expectations? *

Reliability

Now consider all the expectation of "the degree to which the solution will continually operate properly, or will operate in a consistent, flawless manner."

15. How reliable would you expect m-Health solution(s) to be in general? *

Mark only one oval.

	1	2	3	4	5	6	7	
Not at all reliable	◯	◯	◯	◯	◯	◯	◯	Extremely reliable

16. How important would you expect the source credibility of m-Health solutions to be? *

By "source credibility" we mean that the solution is based on any expert information and algorithms.

Mark only one oval.

	1	2	3	4	5	6	7	
Not at all important	◯	◯	◯	◯	◯	◯	◯	Extremely important

17. Please elaborate on all your expectations that you have about the reliability of m-Health solutions. *

____

____

____

____

____

Usefulness

Now consider your expectations of "the degree to which the user trusts that the solution is beneficial for its purpose."

18. How useful would you expect m-Health solutions to be for tracking or managing your chronic pain in general? *

Mark only one oval.

	1	2	3	4	5	6	7	
Not at all useful	◯	◯	◯	◯	◯	◯	◯	Extremely useful

19. How much would you expect that your doctor use information from m-Health solution(s) in office visits? *

Mark only one oval.

	1	2	3	4	5	6	7	
Not at all much	◯	◯	◯	◯	◯	◯	◯	Extremely much

20. How much easier would you expect it to be to follow medical advice, treatmenting guidelines, or any potential exercise routine if you used m-Health solutions? *

Mark only one oval.

	1	2	3	4	5	6	7	
Not at all much	◯	◯	◯	◯	◯	◯	◯	Extremely much

21. How helpful would you expect m-Health solutions to be in reducing your overall concern about your chronic pain? *

Mark only one oval.

	1	2	3	4	5	6	7	
Not at all helpful	◯	◯	◯	◯	◯	◯	◯	Extremely helpful

22. How much would you expect m-Health solutions help you in maintaining your chronic pain? *

Mark only one oval.

	1	2	3	4	5	6	7	
Not at all much	⬭	⬭	⬭	⬭	⬭	⬭	⬭	Extremely much

23. How much time would you expect to save because of using m-Health solutions? *

For instance, do not go to clinique and send your data to your doctor

Mark only one oval.

	1	2	3	4	5	6	7	
Not at all much	⬭	⬭	⬭	⬭	⬭	⬭	⬭	Extremely much

24. How much easier would you expect interacting with health or social care professionals be if you used m-Health solutions? *

Mark only one oval.

	1	2	3	4	5	6	7	
Not at all much	⬭	⬭	⬭	⬭	⬭	⬭	⬭	Extremely much

25. How much more control would you expect m-Health solutions to give you over the activities in your life? *

Mark only one oval.

	1	2	3	4	5	6	7	
Not at all	⬭	⬭	⬭	⬭	⬭	⬭	⬭	Extremely

26. Please elaborate on all the expectations that you have about the usefulness of m-Health solutions in the context of your chronic pain. *

____
____
____
____
____

Other expectations and impressions

27. How much would you expect to use m-Health solutions in the future? *

Mark only one oval.

	1	2	3	4	5	6	7	
Not at all much	◯	◯	◯	◯	◯	◯	◯	Extremely much

28. How fun would you expect m-Health solutions to be? *

Mark only one oval.

	1	2	3	4	5	6	7	
Not at all fun	◯	◯	◯	◯	◯	◯	◯	Extremely fun

29. How much would you expect to recommend m-Health solutions to other people who are in a similar situation to you (they also have chronic pain)? *

Mark only one oval.

	1	2	3	4	5	6	7	
Not at all much	◯	◯	◯	◯	◯	◯	◯	Extremely much

Privacy etc.

30. How much would you expect (anticipate) m-Health solutions to invade your privacy? *

Mark only one oval.

	1	2	3	4	5	6	7	
Not at all much	⬭	⬭	⬭	⬭	⬭	⬭	⬭	Extremely much

31. How safe would you expect that your data collected through your m-Health solutions treated? *

Mark only one oval.

	1	2	3	4	5	6	7	
Not at all safe	⬭	⬭	⬭	⬭	⬭	⬭	⬭	Extremely safe

32. How concerned would you expect to be about your m-Health solution manufacturer having access to your personal data collected via the m-Health solution? *

Mark only one oval.

	1	2	3	4	5	6	7	
Not at all concerned	⬭	⬭	⬭	⬭	⬭	⬭	⬭	Extremely concerned

33. How concerned would you expect to be about your personal data being shared with, e.g., third parties without your permission? *

Mark only one oval.

	1	2	3	4	5	6	7	
Not at all concerned	⬭	⬭	⬭	⬭	⬭	⬭	⬭	Extremely concerned

34. How likely would you expect to donate personal data of different types in order to gain some additional benefits from the use of the m-Health solution, e.g. an additional feature, a PRO version of an app, discounts, etc? *

Mark only one oval per row.

	1- Not at all likely	2	3	4 - Neutral	5	6	7 - Extremely likely
**First name**	⬭	⬭	⬭	⬭	⬭	⬭	⬭
**Surname**	⬭	⬭	⬭	⬭	⬭	⬭	⬭
**Email address**	⬭	⬭	⬭	⬭	⬭	⬭	⬭
**Phone number**	⬭	⬭	⬭	⬭	⬭	⬭	⬭
**Residential address**	⬭	⬭	⬭	⬭	⬭	⬭	⬭
**Iris patterns**	⬭	⬭	⬭	⬭	⬭	⬭	⬭
**Fingerprints**	⬭	⬭	⬭	⬭	⬭	⬭	⬭
**Birth dates and national identification number**	⬭	⬭	⬭	⬭	⬭	⬭	⬭
**Debit/Credit card number**	⬭	⬭	⬭	⬭	⬭	⬭	⬭
**Location data**	⬭	⬭	⬭	⬭	⬭	⬭	⬭

35. Please tell us your reasons for answering the way you did in the previous question? *

____
____
____
____
____

36. Considering the data you produce with the m-Health solution(s) you use, who would you expect to be in control of your data? *

Mark only one oval.

- Only me
- Myself and the m-health solution provider
- The m-health solution provider
- A neutral third-party e.g. a data intermediary established just for data management purposes
- It does not matter

37. Please tell us your reasons for answering the way you did in the previous question? *

____
____
____
____
____

38. What are your thoughts on the future management of your personal health data? *

Feel free to express your thoughts freely. E.g. Who should have access to it? How should it be shared? Where should it be stored? Who should pay for the cost of storage and protection? There are no right or wrong answers.

This content is neither created nor endorsed by Google.

Google Forms
